# Genomic characterisation of novel extremophile lineages from the thalassohaline lake Dziani Dzaha expands the metabolic repertoire of the PVC superphylum

**DOI:** 10.1186/s40793-025-00699-1

**Published:** 2025-05-06

**Authors:** Adrien Vigneron, Lilian A. Cloarec, Céline Brochier-Armanet, Jean Pierre Flandrois, Marc Troussellier, Cécile Bernard, Hélène Agogué, Philippe M. Oger, Mylène Hugoni

**Affiliations:** 1 INSA Lyon, CNRS, UMR5240 Microbiologie Adaptation et Pathogénie, Universite Claude Bernard Lyon 1, 69621 Villeurbanne, France; 2 CNRS, VetAgro Sup, Laboratoire de Biométrie et Biologie Évolutive, UMR5558, Universite Claude Bernard Lyon 1, Villeurbanne, France; 3 MARBEC, IRD, CNRS, Ifremer, Univ. Montpellier, Sète, France; 4 UMR 7245 Molécules de Communication et Adaptations des Microorganismes (MCAM) MNHN-CNRS, Muséum National d’Histoire Naturelle, CP 39, 12 Rue Buffon, 75231 Paris Cedex 05, France; 5 LIENSs, UMR 7266, La Rochelle Université - CNRS, 2 Rue Olympe de Gouges, 17000 La Rochelle, France; 6 Institut Universitaire de France (IUF), Paris, France

**Keywords:** Hypersaline, Extreme environments, Biodiversity, MAGs, Omnitrophota, Elusimicrobia

## Abstract

**Background:**

Extreme environments are useful systems to investigate limits of life, microbial biogeography and ecology, and the adaptation and evolution of microbial lineages. Many novel microbial lineages have been discovered in extreme environments, especially from the Planctomycetota–Verrucomicrobiota–Chlamydiota (PVC) superphyla. However, their evolutionary history and roles in ecosystem functioning and microbiome assemblage are poorly understood.

**Results:**

Applying a genome-centric approach on an 8-year metagenomic timeseries produced from the hypersaline and hyperalkaline waters of Lake Dziani Dzaha (Mayotte), we recovered 5 novel PVC extremophilic candidate lineages from the biosphere of the lake. Sibling to Elusimicrobia and Omnitrophota, these lineages represented novel halophilic clades, with global distributions bounded to soda lakes and hypersaline hydrosystems. Genome mining of these newly defined clades revealed contrasted, but ecologically relevant, catabolic capabilities involved in the carbon, hydrogen and iron/electron cycles of the Dziani Dzaha ecosystem. This also includes extracellular electron transfer for two of them, suggesting metal reduction or potential electron exchanges with other members of the lake community. By contrast, a putative extracellular giant protein with multiple carbohydrate binding domains and toxin-like structures, as observed in virulence factors, was identified in the genome of another of these clades, suggesting predatory capabilities.

**Conclusions:**

Our results provided genomic evidences for original metabolism in novel extremophile lineages of the PVC superphyla, revealing unforeseen implications for members of this widespread and diverse bacterial radiation in aquatic saline ecosystems. Finally, monitoring the in-situ distribution of these lineages through the timeseries reveals the drastic effects of environmental perturbations on extreme ecosystem biodiversity.

**Supplementary Information:**

The online version contains supplementary material available at 10.1186/s40793-025-00699-1.

## Background

Extreme environments such as hydrothermal systems [1–3], polar lakes [4, 5], hypersaline basins [6, 7] and deep underground aquifers [8, 9] are hotspots of unexplored microbial biodiversity with potentially unusual metabolic and adaptive traits. The rapid development of DNA sequencing and computational algorithms has overcome the limitation of microorganism cultivation and now allows the reconstruction and analysis of genomes from uncultured microbial populations [10]. This approach has revealed many new, widespread and highly diversified lineages, collectively referred to as the “microbial dark matter”, expanding our vision of the tree of life [10]. This provides insights into the limits of life, the ecological roles of uncultured microorganisms, and the adaptative mechanisms to extreme conditions [1, 7]. For instance, our knowledge of the taxonomic, genomic, and functional diversities of the enigmatic Patescibacteria (bacterial Candidate Phyla Radiation) or DPANN archaea, has been significantly expanded by genome-centric metagenomics, revealing their ubiquity, abundance and complex entanglement with other microbial lineages [11, 12]. In addition to these major superphyla, which have received much attention, a myriad of new lineages with limited distribution and/or representation have been identified in various biomes, including the most extreme environments [13]. These include Omnitrophota, Elusimicrobia, Auribacterota, Ratteibacteria, Desantisbacteria, Firestonebacteria and Goldbacteria from the superphylum Planctomycetota–Verrucomicrobiota–Chlamydiota (PVC) [9, 14–16]. Despite their relatively low abundances in ecosystems and a presumably small cell size [9, 14, 17], these poorly characterized microorganisms have been found to be hyperactive and to contribute significantly to ecosystem functioning [17], sulfur [18] and/or carbon cycles through complex organic matter degradation and fermentation [14, 19]. Furthermore, Omnitrophota and Elusimicrobia, the best documented lineages of this phylogenetic radiation, display atypical evolution and metabolic capacities [16, 17]. For instance, part of their representatives have been found to be host-associated microorganisms in parasitic or symbiotic interactions, while others are free-living, indicating divergent evolutionary trajectories and contrasted metabolic capabilities [16]. In addition, the genomes of these lineages have been predicted to encode several giant proteins of uncharacterized function that could be involved in interspecies recognition and cell wall degradation [20]. These results argue for an unanticipated implication of these enigmatic and potentially highly active lineages in the global biochemical cycles and in the microbial loop that deserve more attention.

To move in this direction, we present a spatially and temporally genome-resolved metagenomic study conducted on water samples collected between 2014 and 2022 at Lake Dziani Dzaha. This ecosystem is a volcanic maar lake that formed 7000–4000 years ago, trapping oceanic waters that have evolved over time into a warm (~ 30 °C) hypersaline (up to 71 psu) and hyperalkaline (pH 9.4–10) environment [21]. Previous 16S and 18S rRNA gene surveys that characterized its microbial community suggested that microbial activities were initially (in 2014–2015) likely focused on the degradation of the abundant phytoplankton biomass (i.e. the microeukaryote *Picocystis salinarum* and the cyanobacterium *Limnospira fusiformis*) that colonize the water column [22, 23]. However, the seismic crisis that has significantly altered the lake's biogeochemistry since 2018 led to an intense restructuration of its microbiome, inhuming parts of the initial community while awaking novel lineages from the seed bank [24]. These studies also revealed that the lake microbiome had low sequence similarity to known species highlighting its uniqueness and its strong potential for the detection of novel microbial lineages. Focusing on the PVC superphylum, we revealed the undiscovered microbial diversity associated with this poly extreme environment and their potential roles in ecosystem functioning and microbiome assembly. In addition, we disclosed their preferred ecological niches across the multiple geochemical contexts of the lake and the effects of the seismic crisis on these polyextremophile microorganisms.

## Methods

### Study site and samples collection

Lake Dziani Dzaha, located in Petite Terre (Mayotte, Comoros archipelago, latitude 12°46.237′ S, longitude 45°17.315′ E) is a shallow (~ 4 to 5 m deep) volcanic crater lake, with a narrow and 17 m deep pit originating from the chimney of the eruption at the lake’s origin, that has been monitored since 2007 [21]. Until 2017, the geochemistry of the water column was characterized by a strong stability with an oxycline located at ~ 1.5 m, increasing sulfide (up to 6 mM), methane (up to 2 mM) and ammonia (up to 4.5 mM) concentrations with depth, and a salinity gradient with the position of the halocline depending on the precipitation and evaporation levels associated with dry and wet seasons [25, 26]. This geochemical stratification led to the separation of microbial processes between oxic and anoxic niches [27]. However, an active seismicity has recently altered the ecosystem. Underground magmatic movement that began in 2018 led to a strong bubbling of CO_2._ This disrupted the stratifications, averaged the pH (~ 8.1) and introduced oxygen throughout the water column [25]. The mechanical mixing of the water by the advective plume of magmatic gazes emitted from the western slope of pit [25] induces now a deeper and rapidly fluctuating oxygen penetration, creating (micro)-oxic conditions throughout the water column. Consequently, sulfides, methane and ammonia concentrations decreased in the water column, with significant effects on the abundance and composition of the microbial community [24, 25]. Water samples of Lake Dziani Dzaha were collected in the pit during five campaigns (Nov. 2014, Nov. 2015, Nov. 2017, Jun. 2022 and Nov. 2022) at seven (0.25 m, 1 m, 2.5 m, 5 m, 11 m, 15 m and 17 m) discrete depths, covering the contrasted historical and current ecological niches of the lake as previously described [24]. Subsampling and nucleic acid extraction protocols were detailed in a previous study characterizing the microbial community by 16S and 18S rRNA gene sequencing [22].

### Library preparation, sequencing and analysis

Eight metagenomes were previously produced from Nov. 2017 samples (ncbi Bioproject PRJNA1037317), and 42 additional metagenomic libraries, generated from Nov. 2014, Nov. 2015, Jun. 2022 and Nov. 2022 samples, were sequenced using the Illumina NovaSeq 2*150 bp platform by Fasteris company (Plan-Les-Ouates, Swiss), resulting in an average of 1.24 ± 0.17 × 10^8^ reads per samples (Supplementary Table 1). First, sequences were quality filtered using Bbduk v.38.90 [28]. Then, for metagenome assembly, retained sequences were corrected for potential errors using SPAdes v.3.15.4 [29] (–only-error-correction), then pooled and co-assembled using MEGAHIT v.1.2.9 [30]. Read coverage of the contigs was determined using bwa-mem (http://bio-bwa.sourceforge.net). Contigs longer than 2000 bp were binned using MetaBAT-2 [31]. The completeness and contamination levels of the MAGs were assessed using both CheckM v.1.1.5 [32] and CheckM v.2 [33]. The relative abundance of the MAGs across samples was estimated from the contig depths calculated using bwa-mem and extracted using the jgi_summarize_bam_contig_depths script available in MetaBAT-2. Average coverages were calculated per MAGs, then relative abundances were normalized by the number of sequences passing quality filtration per metagenome and multiplied by 1,000,000 to expressed results in reads per million of sequences (RPM). This normalization allows comparison of the MAGs abundance across samples while providing user-friendly numbers. The taxonomic assignment of the MAGs was performed using GTDB-Tk (v.2.4.0, database R220) [34]. In addition, amino acid sequences of ribosomal proteins (rpL2, rpL3, rpL4, rpL5, rpL6, rpL14, rpL15, rpL16, rpL18, rpL22, rpL24, rpS3, rpS6, rpS8, rpS10, rpS17, rpS19) were recovered from the MAGs and reference genomes downloaded from Genbank and the Genome Taxonomy database using HMM profiles. Reference genomes were selected following a three steps strategy. First, we included the closest representative genomes from the genome taxonomy database (GTDB) based on gtdb-tk backbone tree. Then, we compared our genomes on IMG/MER platform and added the closest representatives that were not already included in GTDB. Finally, we included additional genomes from Genbank that were affiliated to Ca. Auribacterota. Dataset was curated for completeness and contamination then only MAGs with at least 70% of the 17 ribosomal proteins were considered. These sequences were aligned with using Mafft v7.511 [35]. Resulting multiple alignments were concatenated in a large supermatrix combining 166 sequences and 6413 amino acid positions. A maximum likelihood phylogenetic tree was inferred using IQ-TREE v3 [36] using model LG + F + I + G4 as determined by IQ-TREE. The branch robustness was measured with the fast bootstraps procedure implemented in IQ-TREE (1000 replicates) and the SH-aLRT test (1000 replicates). The resulting tree was visualized using iTOL v.6 [37]. Pairwise average amino-acids identity (AAI) percentages between MAGs were calculated using the Genome-based distance matrix calculator [38].

Open reading frames (ORFs) of the MAGs were identified using Prodigal v.2.6.3 [39], then the functional annotation was carried out using KofamScan with the KEGG database v110.0 [40]. To consolidate annotation results, MAGs were also processed locally through NCBI’s prokaryotic genome annotation pipeline (PGAP v6.6 2023–10-03.build7061) [41] and screened with custom HMM profiles collected from METABOLIC v.4 [42], FeGenie [43] and metabolisHMM [44]. The results were manually checked for the presence of specific pathways. Predicted protein sequences were also compared against the CAZY database using dbCAN3 [45], and glycoside hydrolase genes were analyzed to infer the catabolic potential of the MAGs. Hydrogenase sequences were classified using HydDB [46]. Individual phylogenies of key genes (hydrogenase, *ndh2*, *eetA*, *hxlA*) were constructed as described below to confirm their identity. The protein sequences of these genes were compared against the NCBI non-redundant protein database using BLASTP. Best hits of Blast (10 per query) as well as reference sequences (reviewed Uniprot entries for *hxlA*, *ndh2* and *eetA* genes, hydrogenase sequence database from HydDB) were downloaded. Amino-acid sequences were aligned using Mafft [35] and maximum-likelihood trees were calculated with IQTREE v.3 with 1000 bootstraps and 1000 SH-aLRT [36].

### Large protein analysis

Proteins larger than 5000 amino acids with no characterized function were recovered from the MAGs and reference genomes then analyzed in detail. Domains were identified using the NCBI RPS-BLAST against the Conserved Domain Database, InterProScan 5.61–93.0 with Interpro database v.93 [47] and Phyre2 [48]. Presence of signal peptide was determined by SignalP 6.0 [49] and putative localization of the proteins and the number of transmembrane helixes by DeepTMHMM v1.0.24 [50] and DeepLocPro v1 [51].

## Results and discussion

### Lake Dziani Dzaha hosts novel lineages of extremophiles from the PVC superphylum

**Fig. 1:**
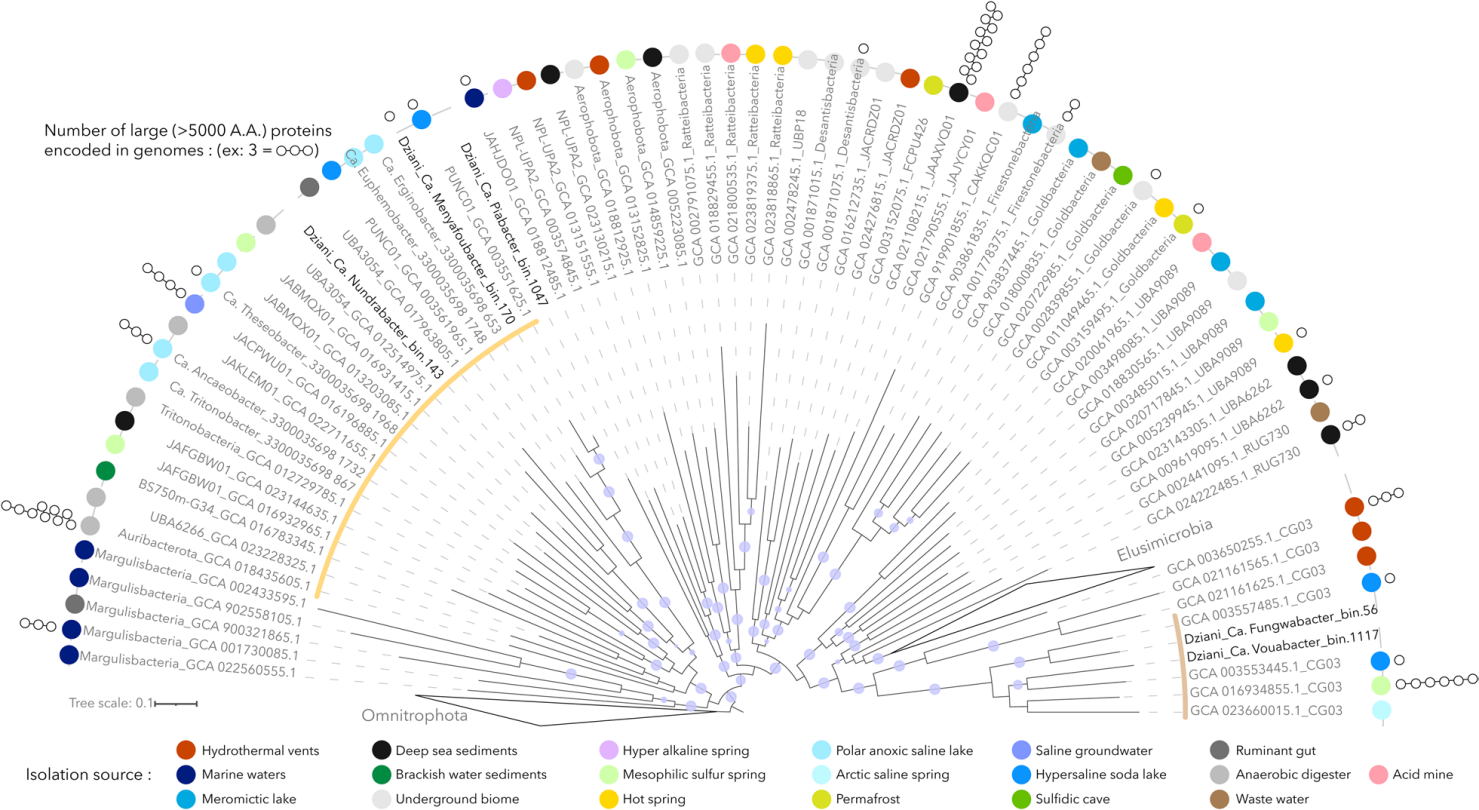
Maximum likelihood tree of the PVC subgroups analysed in this study. The tree was calculated based on the concatenated alignment of 17 ribosomal proteins and rooted with the Omnitrophota phyla. Only bootstraps > 90% were represented by purple dots on the branches. Colored dots reflect the biome of origin where reference genomes were recovered. White circles at the end of the branches indicate the number of putative large (> 5000 amino-acids) proteins identified in genomes. Novel genomes (n = 5) are labeled in bold. Yellow and brown arcs indicate the Auribacterota and Shingomicrobia clades, respectively

After binning of the contigs reconstructed from the co-assembly, a total of 1185 unique MAGs was recovered, including 582 medium- to high-quality MAGs (> 70% completeness and < 5% contamination). Taxonomic assignment of the MAGs performed using the GTDB-Tk pipeline indicated that 16 of the 582 good-quality MAGs belonged to 11 uncharacterized phyla, revealing that the poly-extreme Lake Dziani Dzaha harbors an unexplored genomic diversity of microorganisms. Although 16S rRNA genes were not included in the MAGs, the phylogenomic analysis of ribosomal proteins indicated that a third (n = 5) of these uncharacterized MAGs belonged to the Planctomycetes-Verrucomicrobia-Chlamydia superphylum, as siblings of the Omnitrophota and Elusimicrobia clades (Fig. 1 and supplementary Table 2).

**Fig. 2:**
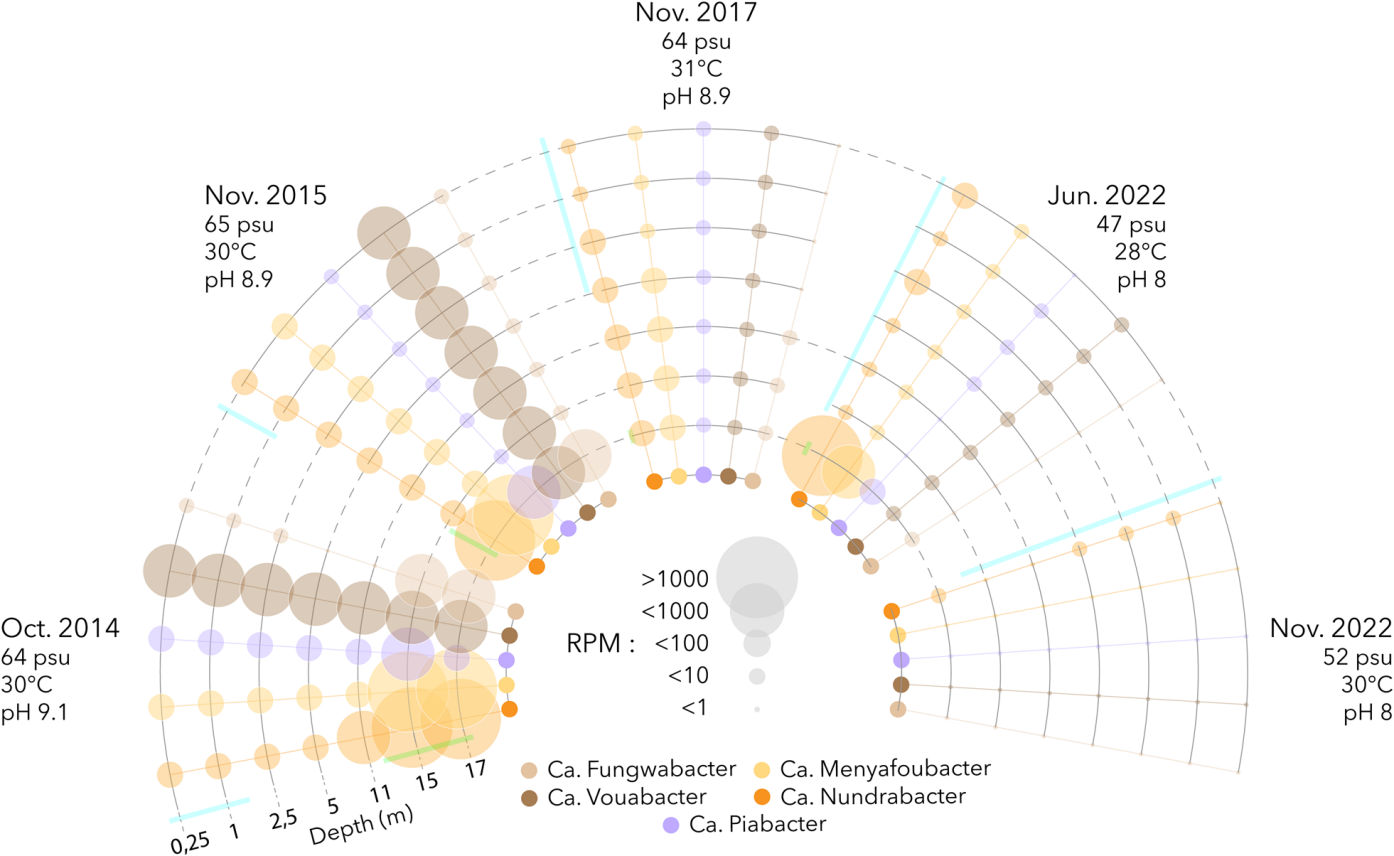
Depth and seasonal distribution of the MAGs. Size of the dots represents the relative proportion (RPM: reads per million of sequences) of each MAG in the dataset. Blue and green bars localise the position of the oxic and sulfidic zones in the water column. Average salinity, temperature and alkalinity throughout the water column during the different sampling periods are indicated along the dates

Two of them, which we named Ca. Menyafoubacter (bin.170) and Ca. Nundrabacter (bin.143) (Menyafou and Nundra meaning “destructive” and “deep”, respectively, in Shimaore, one of the languages of the indigenous people of Mayotte) branched out into the poorly resolved Auribacterota phyla, close to Ca. Erginobacter and Ca. Euphemobacter that were recovered from the anoxic saline waters of Lake Ace (Antarctica) [4]. In contrast, Ca. Fungwabacter (bin.56) and Ca. Vouabacter (bin.1117) (Fungwa and Voua meaning “trapped” and “rainfall”, respectively, in Shimaore) belonged to the uncharacterized candidate phyla CG03, and formed a distinct (pairwise amino acid identity < 50% with other phyla, Supplementary Table 3), monophyletic and cohesive group with genomes recovered from anoxic saline lakes, which we propose to name Shingomicrobia; “Shingo” meaning salty in Shimaore. Finally, bin.1047, which branched deeply in the phylogenomic tree and shared a low (43.91%) average amino acid identity with the closest reference genome (JAHJDO01 GCA018812485) may correspond to a novel class in an as yet undefined phylum, for which we propose the name of Ca. Piabacter (“Pia” meaning “novel” in Shimaore). Analysis of the environmental niches in which reference genomes were recovered indicated that, with the exception of Ca. Nundrabacter which forms a taxonomically distinct group with genomes recovered from anoxic saline waste digesters, all novel lineages cluster with genomes originating from hypersaline soda lakes and polar anoxic saline lakes [4, 6, 18], with environmental conditions very similar to Lake Dziani Dzaha. Furthermore, the depth and seasonal distribution of the MAGs in the water column of lake Dziani Dzaha indicated a niche preference for the highly sulfidic (4–6 mM of H_2_S), anoxic environments and extreme salinity (60–70 psu) and alkalinity (pH 10) that characterized the bottom of the lake in 2014 and 2015 [22] and to a lesser extend in June 2022 (Fig. 2). For example, up to 2750 sequences per million of sequenced reads were detected in 2014 for Ca. Menyafoubacter. In contrast, the coverage of the MAGs decreased since 2017, reaching less than 1 sequences per million of sequenced reads in 2022, possibly corresponding to relict DNA. Altogether, these results suggest that these lineages form novel groups of extremophiles adapted to hypersaline and alkaline environments and illustrates that extreme environments are sources of an unexplored biodiversity.

**Fig. 3:**
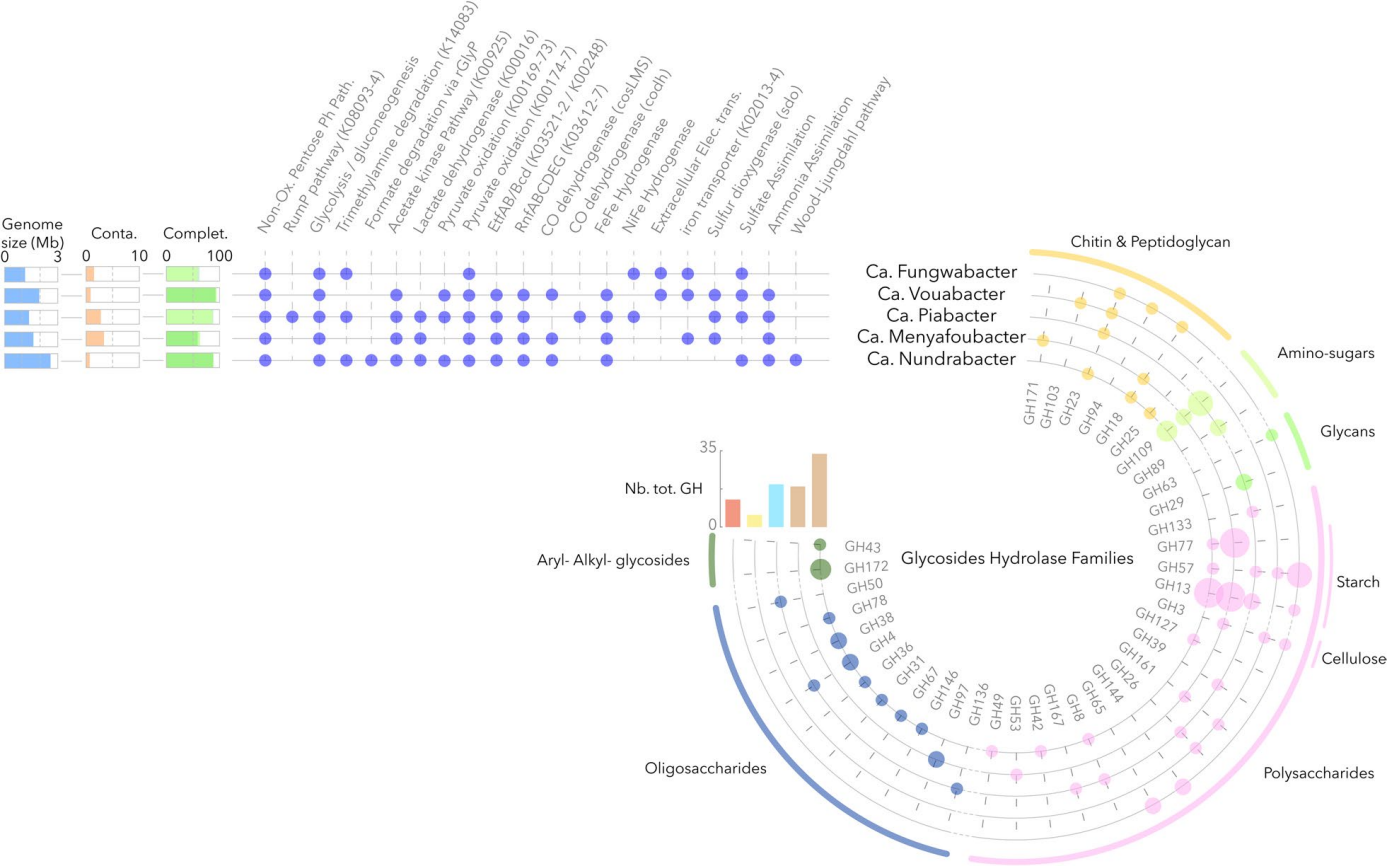
Genome content and metabolic prediction of the MAGs. Genome size, contamination and completeness were determined using CheckM1 and CheckM2. The presence of metabolic pathways was assumed when genes were identified using both KofamScan and PGAP functional annotations. Extracellular electron transfer locus detection was performed using FeGenie and the sets of glycoside hydrolases were predicted by dbCAN3. Size of the dot in glycoside hydrolase circles indicates the number of copies of the gene (max = 5), and colors indicate the potential substrate families

Although, these bacteria were relatively rare in the Lake Dziani Dzaha microbial community, ranking from 115 to 470th in relative abundance among the 582 medium- to high-quality MAGs recovered from Lake Dziani Dzaha, minority lineages could have major ecological roles in ecosystems [52]. Genomes mining suggested a strictly anaerobic lifestyle without respiratory cytochromes (Fig. 3), which is consistent with the depth distribution of the lineages (Fig. 2) and genomes mining of the sibling groups Omnitrophota [17] and Elusimicrobia [16]. However, despite their taxonomic branching within the PVC superphylum, a large dissimilarity in gene content (Bray–Curtis similarity < 0.57) was detected between genomes, suggesting contrasted metabolic capabilities and ecological functions and illustrating the large genomic diversity within the PVC superphylum.

### Extracellular electron transfer in Shingomicrobia (non-thermophilic CG03) lineages

**Fig. 4:**
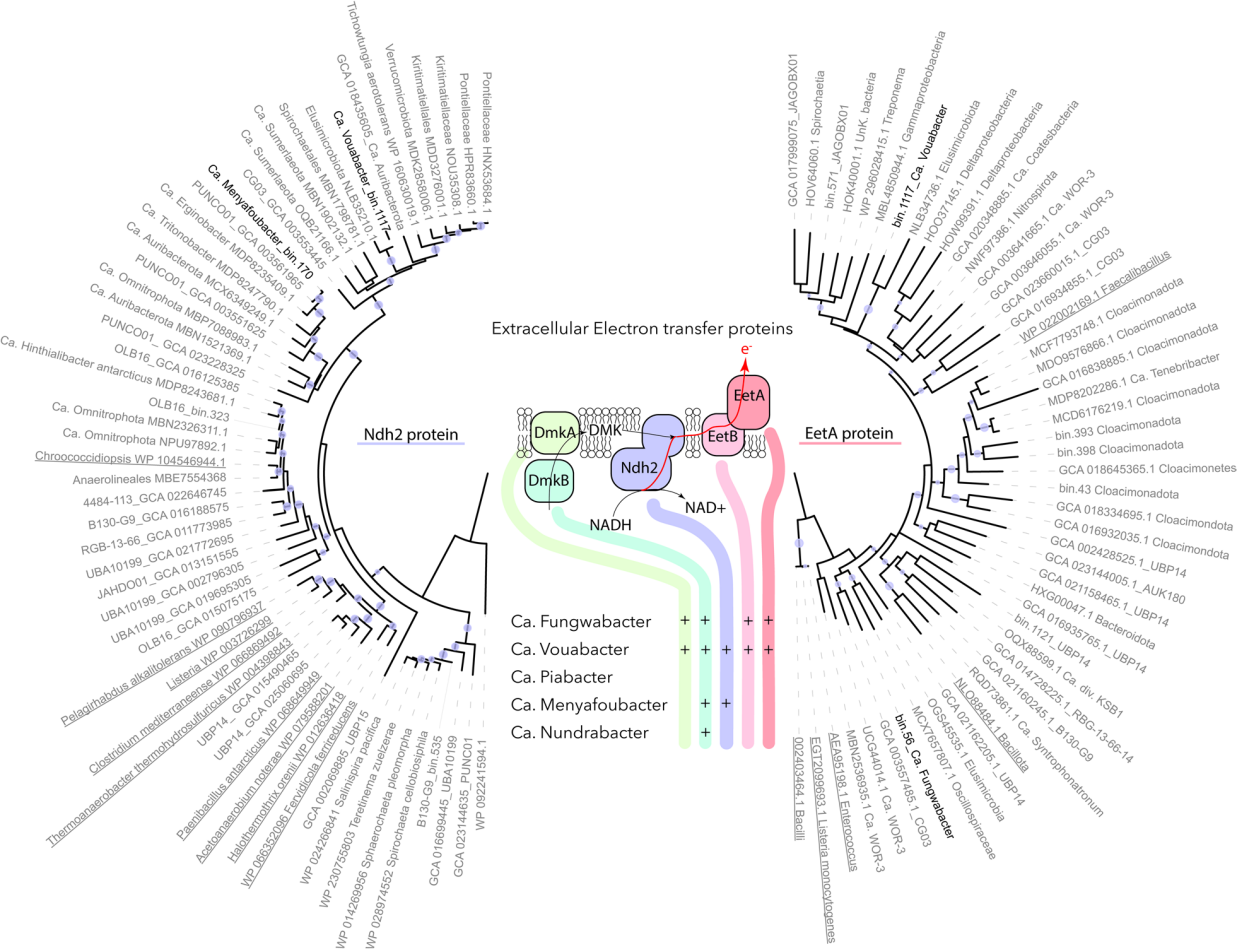
Composition of the extracellular electron transport complex in the MAGs and phylogenetic trees of Ndh2 and EetA protein sequences. MAGs recovered from Lake Dziani Dzaha are shown in bold and lineages with probable functional extracellular electron transport complex are underlined. Only bootstraps > 90% are represented by purple dots on the branches

Genes encoding group A3 FeFe bifurcating hydrogenases (Fig. 3 and Supplementary Fig. 1), RnfABCDEG and EtfAB/Bcd complexes were found in the genomes of Ca. Vouabacter, Ca. Piabacter, Ca. Menyafoubacter and Ca. Nundrabacter (Fig. 3), indicating that flavin-based electron bifurcation systems are largely represented in the genomes of these lineages, as observed in the sister phyla Elusimicrobia [16]. These systems, often found in strictly anaerobic bacteria, provide these lineages with mechanisms for energy conservation, intracellular redox balance and hydrogen cycling, allowing exergonic reactions in the cells [53]. Interestingly, a flavin-based extracellular electron transfer (FLEET) locus [54] was also detected in the taxonomically close Ca. Vouabacter and Ca. Fungwabacter lineages (Fig. 4). These genes were also identified in the genomes of the non-thermophilic CG03, which clustered with these lineages within the Shingomicrobia clade, supporting the monophyly of this clade (Fig. 4), as well as in the recently described phyla of Candidatus Effluviviacota that also branched within the PVC superphyla [55]. Although further research is needed to explore the functional implications of these findings and the potential interactions between these genes and other metabolic pathways. The detection of the FLEET locus indicates that in addition to the Rnf complex and hydrogenases, Ca. Vouabacter and Ca. Fungwabacter lineages could transfer electron extracellularly for energy production [54, 55]. Soluble iron (II) and ferrihydrite are supplied to the Lake Dziani Dzaha by the weathering of the crater [21]. These compounds have been found to accept electrons from FLEET [56], supporting an extracellular iron reduction metabolism for these microorganisms. This reaction could be particularly advantageous in Lake Dziani Dzaha, where elevated H_2_S/HS^−^ concentrations, that were measured at the beginning of the survey (2014–2015) [24], form conductive iron sulfides that accelerate and extend the process through distant ecological niches [57]. Extracellular electron transfer has previously been associated with syntrophic relationships, such as in anaerobic methane oxidizing consortia, where sulfate-reducing bacteria act as electron acceptors [58]. Therefore, unidentified members of the community could also potentially be substitute electron acceptors for Shingomicrobia in an original syntrophic relationship. Alternatively, this pathway could enhance ecological fitness by contributing to the cellular NADH/NAD^+^ and proton redox balance, or by enhancing iron bioavailability under anoxic conditions [59], which would be assimilated via the iron complex transport system also identified in the genomes of these lineages (Fig. 3). Phylogenetic trees of the specialised type II NADH dehydrogenase Ndh2 (bin.1117_001767) and the EetA protein (bin.56_001019, bin.1117_001550) involved in the transmembrane apparatus revealed a taxonomic proximity with sequences related to Elusimicrobia, Auribacteria, Kiritimatiellales, Tichowtungiia and Cloacimonadota, supporting a broader but paraphyletic distribution of the FLEET locus within the PVC superphylum (Fig. 4).

### Carbon monoxide, amines and formaldehyde assimilation by Ca. Piabacter

**Fig. 5:**
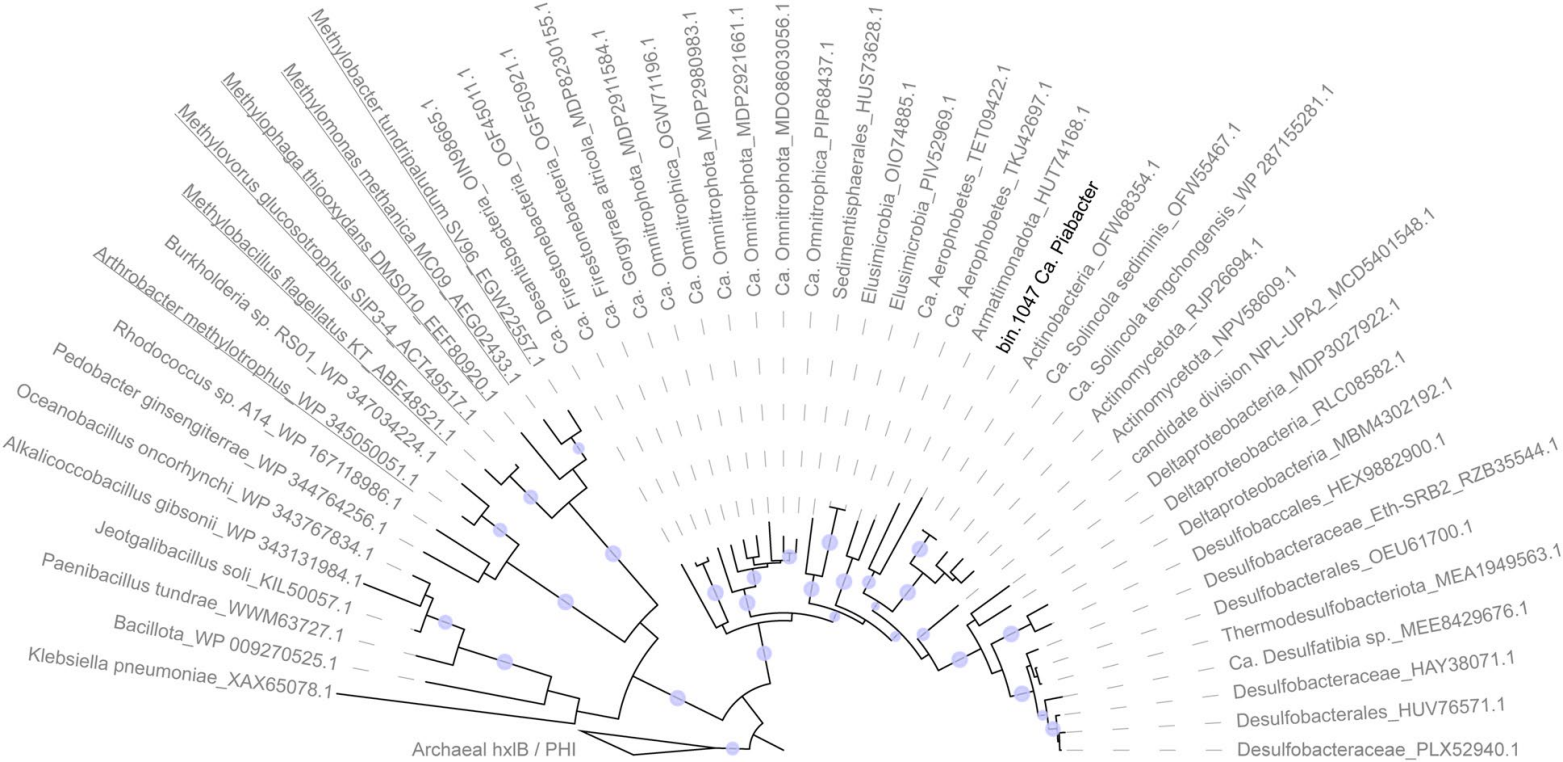
Phylogenetic tree of the HxlB/PHI protein sequences. MAG of Ca. Piabacter recovered from Lake Dziani Dzaha is shown in bold and genomes from C_1_ oxidatizing lineages are underlined. Only bootstraps > 90% are represented by purple dots on the branches. Tree was rooted with archaeal Hxlb sequences

The study of Ca. Piabacter MAG revealed a specialization in the fermentation of small carbonated molecules, including (tri)methylamines and amino-sugars. A CO dehydrogenase (CODH) and acetyl-CoA synthase (ACS) complex, coupled with [NiFe] hydrogenase group 4c was also detected, indicating a carbon monoxide metabolism. Although minimal, this complex provides an efficient primary strategy for energy production [60] and an escape from competition. In addition, the potential for formaldehyde assimilation through the *hxlAB* operon, encoding the 3-hexulose-6-phosphate synthase (HPS) and 6-phospho-3-hexuloisomerase (PHI), was also detected (bin.1047_000919 and 1047_000920). These genes, which are part of the ribulose monophosphate (RuMP) pathway, are usually detected as a one-carbon assimilation pathway in aerobic methylotrophic bacteria and as a detoxification process in non-methylotrophic aerobes [61]. Similar but taxonomically distant genes are also found in anaerobic archaea and function as an alternative pentose phosphate pathway [62]. However, the presence of this pathway in an anaerobic bacterium is questionable. BlastP of the *hxlAB* operon identified in Ca. Piabacter against the NCBI database and phylogenetic analysis of *hxlB* showed similarities with sequences recovered from sibling phyla of Ca. Omnitrophota, Ca. NPL-UPA2, Ca. Firestonebacteria, Desantisbacteria, Ca. Aerophobus, which formed a distant cluster of environmental sequences with some Desulfobacteraceae and Actinobacteria representatives (Fig. 5). Although experimental validation is required because neighboring sequences in the phylogenetic tree are uncharacterized, the presence of the *hxlAB* genes and the subsequent pathway for the degradation and assimilation of the fructose-6-phosphate supports the presence of an unforeseen formaldehyde assimilation/detoxification in anaerobic uncultivated lineages of the PVC superphylum.

### Extended catabolic arsenal of Ca. Nundrabacter

Among the PVC MAGs from this study, Ca. Nundrabacter has the largest genome with up to 2.6 Mb and 2045 predicted coding genes. Consistently, an extended metabolic repertoire was detected, including a mixotrophic lifestyle with both CO_2_ fixation potential via the Wood-Ljungdhal pathway and fermentations with lactate, acetate and potentially hydrogen as end products. Formate utilisation via the reductive glycine pathway was also identified, including genes encoding the formate tetrahydrofolate (THF) ligase (bin.143_002017), the methenyl-THF cyclohydrolase and dehydrogenase (bin.143_001550), and a complete glycine cleavage system (bin.143_001051-53) [63]. Trimethylamine degradation potential (bin.143_001855) was also detected in Ca. Nundrabacter providing an additional source of carbon and nitrogen. A large catabolic arsenal was also predicted in Ca. Nundrabacter genome with up to 22 different (35 in total) subfamilies of glycoside hydrolases and 23 carbon-binding modules targeting various polysaccharides (rhamnose, sucrose, xylan, arabinan, glucan, mannan), reserve molecules (glycogen, glucosylglycerate, starch), chitin, peptidoglycan, and aryl- and alkyl-glycosides (Fig. 3). Similar extended degradation capabilities were also identified in the closest references genomes, named UBA3054 in the Genome Taxonomy database, that were recovered from ruminant gut and anaerobic digesters, suggesting that they form a distinct clade of anaerobic recyclers of complex organic matter within the phylum Auribacterota.

### Novel extracellular large catabolic proteins in Menyafoubacteria

**Fig. 6:**
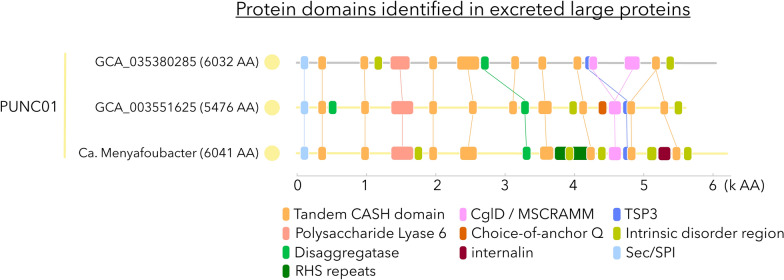
Characterization of the putative large proteins identified in the MAG of Ca. Menyafoubacter and related reference genomes of the PUNC01 clade. Sec/SPI: secretory signal peptides transported by the Sec translocon and cleaved by signal peptidase I; CASH: carbohydrate-binding/sugar hydrolysis beta-helix; RHS: rearrangement hotspot; MSCRAMM: microbial surface components recognizing adhesive matrix molecules; TSP3: bacterial thrombospondin-3

Numerous lineages in the PVC superphyla have been predicted to contain large to giant genes, suggesting the production of massive proteins (up to 85,800 amino acids) [20]. This is particularly relevant in the PVC clades of Omnitrophota [17], Elusimicrobia and CG03, where giant genes are common in the genomes [20]. While the post-transcriptional integrity of such large proteins remains unclear, the presence of multiple transmembrane helixes and carbohydrate degradation domains has been interpreted as massive surface weapons for predatory bacteria [17, 20] or novel extracellular cellulosome-like structures [15]. Screening of the predicted proteomes revealed the presence of large proteins (> 5000 amino acids) (Fig. 1). However, the taxonomic distribution of large proteins was patchy, maybe due to methodological differences for the assembly and binning of genomic data. Among the MAGs recovered from Lake Dziani Dzaha, only Ca. Menyafoubacter harbored a predicted large (~ 6000 aa) protein (bin.170_000073) with a standard secretory signal peptide and no transmembrane helix suggesting an extracellular localization (Fig. 6).

The large protein of Ca. Menyafoubacter has several tandem carbohydrate-binding/sugar hydrolysis beta-helix (CASH) domains similar to pectin-lyase beta-helix and a large polysaccharide lyase 6 (e.g. alginate lyase) domain, suggesting a carbohydrate-degrading potential, focused on green algal cells. A disaggregase region, potentially inducing dissociation of cell aggregates [64] and various domains found in virulence factors such as in Rhs toxin that induces apoptosis of neighboring cells [65, 66], CglD-type adhesin and internalin domains were also identified in the protein, potentially favorizing its adhesion to other cells [67]. In addition, intrinsically disordered regions that allow structural flexibility [68] were also detected. Very similar proteins were also identified in taxonomically close MAGs recovered from a soda lake of the Kulunda steppe [6] (PUNC01 GCA_003551625) and anoxic saline waste digesters (PUNC01 GCA_035380285), supporting the distribution and structure of this large protein within this extremophile clade. Taken together, these results suggest that Ca. Menyafoubacter and related lineages could potentially catalyze the degradation of plant and/or green alga complex. The vegetation around Lake Dziani Dzaha and in the Kulunda steppe are highly contrasted. However, both ecosystems host a large population of the trilobed picoalgae *Picocystis salinarum* [69]*,* which could potentially be the target of this large extracellular protein.

### Roles in ecosystem functioning

Members of the PVC superphylum have been found to be involved in numerous biochemical transformations, such as in the nitrogen [70, 71], methane [72, 73] and sulfur [18, 74, 75] cycles. Our results indicate that the newly defined lineages are likely to be involved in the carbon, hydrogen and iron/electron cycles in hypersaline and alkaline ecosystems, expending our knowledge of the metabolism of the PVC members. In addition, these lineages may contribute to the recycling of the biomass as potential primary degraders or predators, extending previous observations from some members of the sister taxon Omnitrophota [17] and suggesting an unforeseen implication in the microbial loop of extreme environments. Although, these genomic investigations of the dark microbial matter lineages related to PVC superphylum require experimental and functional validations, they revealed a large metabolic diversification among the extremophilic lineages of this phylogenetic radiation, possibly related to niche differentiation and substrate availability and revealed contrasted interactive behaviors with other members of the community. Fluctuations in their distribution and abundance along the water column over time highlighted their high sensitivity to the “moderation” of the environmental conditions in the lake (e.g. oxygenation, decrease in pH and sulfide concentration). Although all MAGs contained antioxidant enzymes (e.g. peroxiredoxin, super oxide dismutase or reductase, glutaredoxin), suggesting a relative tolerance to oxic stress, the changes in environmental conditions seemed to have been fatal for these lineages, confirming their strictly extremophilic lifestyle, and highlighting the major consequences of environmental perturbations for biodiversity in extreme environments.

## Supplementary Information

Additional file 1.

Additional file 2.

Additional file 3.

## Data Availability

Data availability Geochemical data has been previously published 25. The raw metagenomic reads produced from Lake Dziani Dzaha are available in NCBI SRA database under BioProjects PRJNA1037317 and PRJNA1222159 (description of the Bioprojects is available in Supplementary Table 1). Nucleotide and amino acid sequences of the MAGs as well as annotation files are freely available in figshare: 10.6084/m9.figshare.26347213.
