# Supplementary material for: Genomic characterisation of novel extremophile lineages from the thalassohaline lake Dziani Dzaha expands the metabolic repertoire of the PVC superphylum
Source: Environ Microbiome. 2025 May 6;20:48. doi: 10.1186/s40793-025-00699-1 (PMC12057145; doi:10.1186/s40793-025-00699-1)
Supplement: Supplementary file 1 — Additional file 1. [file 40793_2025_699_MOESM1_ESM.docx]

**Supplementary Information**

Supplementary Table 1: SRA metadata table with accession numbers of each metagenome analysed in this study.

Supplementary Table 2: Detailed description of the MAGs analysed in this study. Completeness, contamination, genome size, GC content, coding density and number of predicted proteins were determined using CheckM2. Taxonomy and RED value were obtained using GTDB-tk pipeline. Relative abundance of the MAGs across samples was determined using coverage data calculated using bwa-mem.

Supplementary Table 3: Average amino acid identity matrix of CG03 phyla. Names in bold indicate lineages delineating Shingomicrobia clade.

| AAI CG03 | Ca. Vouabacter | Ca. Fungwabacter | GCA_003553445.1 | GCA_016934855.1 | GCA_023660015.1 | GCA_003557485.1 | GCA_021161625.1 | GCA_021161565.1 | GCA_003650255.1 |
| --- | --- | --- | --- | --- | --- | --- | --- | --- | --- |
| Ca. Vouabacter bin.1117 | **1.00** | **0.50** | **0.53** | **0.51** | **0.51** | **0.51** | 0.42 | 0.41 | 0.43 |
| Ca. Fungwabacter bin.56 | **0.50** | **1.00** | **0.54** | **0.50** | **0.48** | **0.61** | 0.41 | 0.42 | 0.43 |
| GCA_003553445.1 | **0.53** | **0.54** | **1.00** | **0.52** | **0.51** | **0.54** | 0.41 | 0.42 | 0.44 |
| GCA_016934855.1 | **0.51** | **0.50** | **0.52** | **1.00** | **0.53** | **0.51** | 0.42 | 0.43 | 0.45 |
| GCA_023660015.1 | **0.51** | **0.48** | **0.51** | **0.53** | **1.00** | **0.51** | 0.42 | 0.42 | 0.44 |
| GCA_003557485.1 | **0.51** | **0.61** | **0.54** | **0.51** | **0.51** | **1.00** | 0.42 | 0.42 | 0.44 |
| GCA_021161625.1 | 0.42 | 0.41 | 0.41 | 0.42 | 0.42 | 0.42 | 1.00 | 0.59 | 0.51 |
| GCA_021161565.1 | 0.41 | 0.42 | 0.42 | 0.43 | 0.42 | 0.42 | 0.59 | 1.00 | 0.49 |
| GCA_003650255.1 | 0.43 | 0.43 | 0.44 | 0.45 | 0.44 | 0.44 | 0.51 | 0.49 | 1.00 |

Supplementary Figure 1: Phylogenetic tree of FeFe hydrogenase genes on the alignment of amino acid sequences. Only bootstraps > 90% were represented by purple dots on the branches.

**
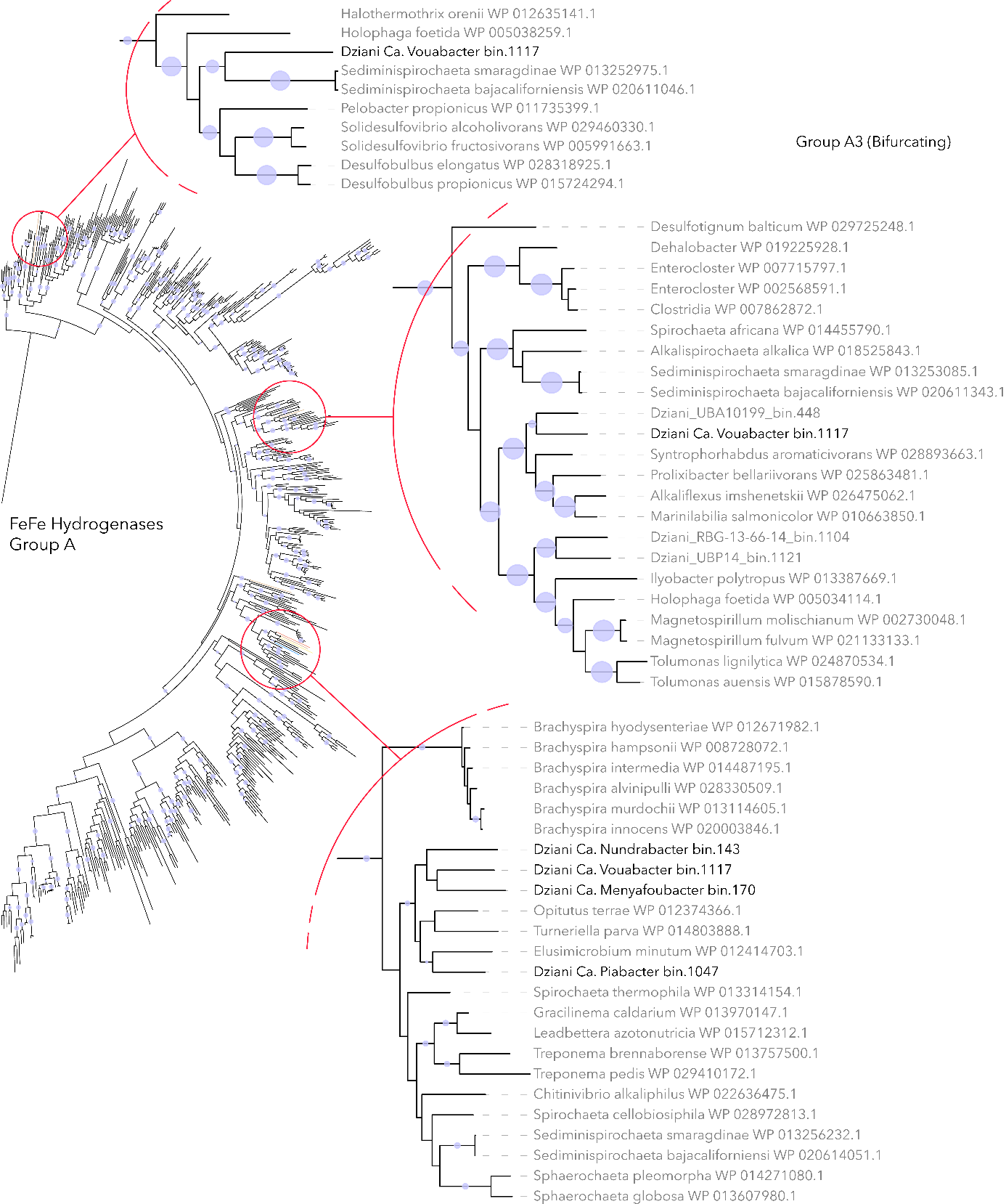
**
